# Layer-by-layer films of polysaccharides modified with poly(N-vinylpyrrolidone) and poly(vinyl alcohol)

**DOI:** 10.1016/j.heliyon.2021.e08224

**Published:** 2021-10-21

**Authors:** Kanstantsin S. Livanovich, Anastasiya A. Sharamet, Anna N. Shimko, Tatsiana G. Shutava

**Affiliations:** a Institute of Chemistry of New Materials, National Academy of Sciences of Belarus, Minsk, Belarus; b Center for Analytical Spectral Measurements, B.I. Stepanov Institute of Physics, National Academy of Sciences of Belarus, Minsk, Belarus

**Keywords:** Layer-by-layer assembly, Chitosan copolymer, Protein adsorption, Poly(N-vinylpyrrolidone), Poly(vinyl alcohol)

## Abstract

N-grafted copolymers of chitosan (460 kDa) with poly(N-vinylpyrrolidone) (2.4 kDa) or poly(vinyl alcohol) (2.0 ​kDa) as side chains were synthesized. Depending on the polymer-to-chitosan mass ratio the degree of amino group substitution with side chains in chitosan backbone was varied in the range of 0.01–0.33. Layer-by-layer films consisted of copolymers and dextran sulfate as polyanion were obtained. Thickness, hydrophilicity, and morphology of the films were investigated using QCM, UV-vis spectrophotometry, AFM, and contact angle measurements. The obtained films show enhanced protein-repellent properties in fetal bovine serum medium. The mass of adsorbed proteins on LbL films based on copolymer with a degree of substitution of 0.2 decreases by 50 ​% compared to unmodified chitosan. Protein-repellent properties of copolymer-based films are common for LbL films of grafted chitosan copolymers and depend on hydrophilic side chain density on the surface.

## 1 Introduction

Formation of protein-resistant coating is an essential topic in biomimetic surface engineering. Nonspecific protein adsorption on artificial surfaces of catheters, implants, nanoparticles, etc. plays an important role in the innate immune system response to the foreign objects stimulating complex of inflammatory or allergic reactions, activating the complement system [1]. To avoid the immune response the surfaces are modified through plasma etching, chemical vapor deposition or phy­­sisorption of biomimetic species (proteins, cells, hydrogels, polymers) ​[1, 2, 3].

One of the most effective methods to reduce the immune response on artificial implants is the chemisorption of polyethylene glycol (PEG) chains to form the hydrophilic brushes preventing nonspecific adsorption [1, 4]. It has been shown that a dextran (DEX) layer also effectively suppresses biofouling [5].

Aiming to high aggregative stability in biorelevant solutions poly(N-vinylpyrrolidone) (PVP) and poly(vinyl alcohol) (PVA) are often used to modify surface of nanocarriers. To prepare stable copolymer-based nanoparticles for atorvastatin, PVP 40 kDa was admixed to polymerizing mixture of methacrylic and acrylic acids [6]. The addition of PVP 10 ​kDa to ammonium hydroxide solution prior the sol-gel synthesis produces stable polymer-capped SiO_2_ nanoparticles for adsorption of bilirubin [7]. Chitosan-graft-PVP copolymer was shown to be an effective DNA carrier [8]. Carboxylated by NaOH treatment PVP was used in preparation of stable silver nanoparticles (AgNP) conjugated with cytokine that show high anti-inflammatory effectiveness in vitro [9]. PVA effectively stabilizes nanooxide AST 50 (Al_2_O_3_–SiO_2_–TiO_2_) [10]. The capping with PVP remarkably improves the stability of hybrid AgNP in protein-containing environments especially in bovine serum albumin (BSA) solutions [11].

Similar to other hydrophilic polymers, the level of surface-protein interaction can be addressed through polyvinyl coating. PVP-coated metallic nanoparticles exhibit much weaker interactions with BSA than citrate-coated gold and silver ones [12]. Preadsorption of polymers PEG or PVP which have electron-donor sites C–O–C and C–N–(C=O)-, respectively, reduced subsequent adsorption of gelatin or ovalbumin onto silica surface in an amount-dependent manner. However, pre-adsorption under the same conditions of PVA which has electron- and proton-donor OH groups led to a smaller reduction in the maximum gelatin adsorption onto PVA/silica [13]. The PVA-modified surface showed good resistance to the adsorption of human serum albumin (HSA), and there was no evidence of HSA displacing the PVA [14].

On the other hand, it was reported that the incorporation of PVP greatly increases the amount of BSA that can be immobilized onto the porous silica surface [15]. Coating with PVP induces differences in the composition of “protein corona” on AgNP between particles of identical chemical composition and size [16] which, in turn, affects their cellular uptake and macrophage response [17].

The main withdraws of direct coating of surfaces with PVP or PVA are the necessity of the specific pretreatment of substrate and the polymers to be capped in order to form covalent or electrostatic bonds rather than week hydrogen bonding, the inability to control the amount of physisorbed polymer, and possibility of its displacement by proteins.

Layer-by-layer (LbL) assembly approaches surface modification in a controllable manner using strong electrostatic interaction as the main driving force between sequentially and alternately adsorbing polyanion and polycation to form a multilayer film [18]. Properties of LbL films, such as thickness, morphology, permeability etc. are controlled by pH, temperature, polyelectrolyte structure, and solvent properties [19]. The application of LbL modified surfaces has been investigated for drug encapsulation [20], for prolonged release preparations [21, 22] implant protection [23], and tissue engineering [24].

Electrostatic adsorption of graft copolymer of polyelectrolyte with hydrophilic side chains is a promising way to improve surface biocompatibility [25, 26]. Adsorption of graft copolymers of poly-L-lysine with side chains of poly(ethylene glycol) or dextran effectively decreases the inflammation activity and nonspecific protein adsorption [26, 27]. The proposed mechanism of graft copolymer action involves formation of hydrophilic layer of touching each other side chains [4]. Such layer prevents cell or bacteria adhesion as well as protein adsorption due to both hydrophilic surface and steric repulsion. The effectiveness of inhibiting action of copolymers is regulated by the surface density of side chains. The ratio of average distance between the side chains on the surface (L) to macromolecular radius of gyration (R_g_) is used to quantify polymer surface density.

DEX or PEG as side chains decrease adsorbed mass of fetal bovine serum proteins on LbL films made up from their graft copolymers with chitosan and dextran sulfate (DS) as polyanion [28]. The decrease in protein adsorption can be attributed to either specific structure of copolymer or the structure of LbL films. So, it is interesting to investigate if protein repelling properties are characteristic for LbL films of graft copolymers with different side chains and how their surface density effects it.

**Figure 1:**
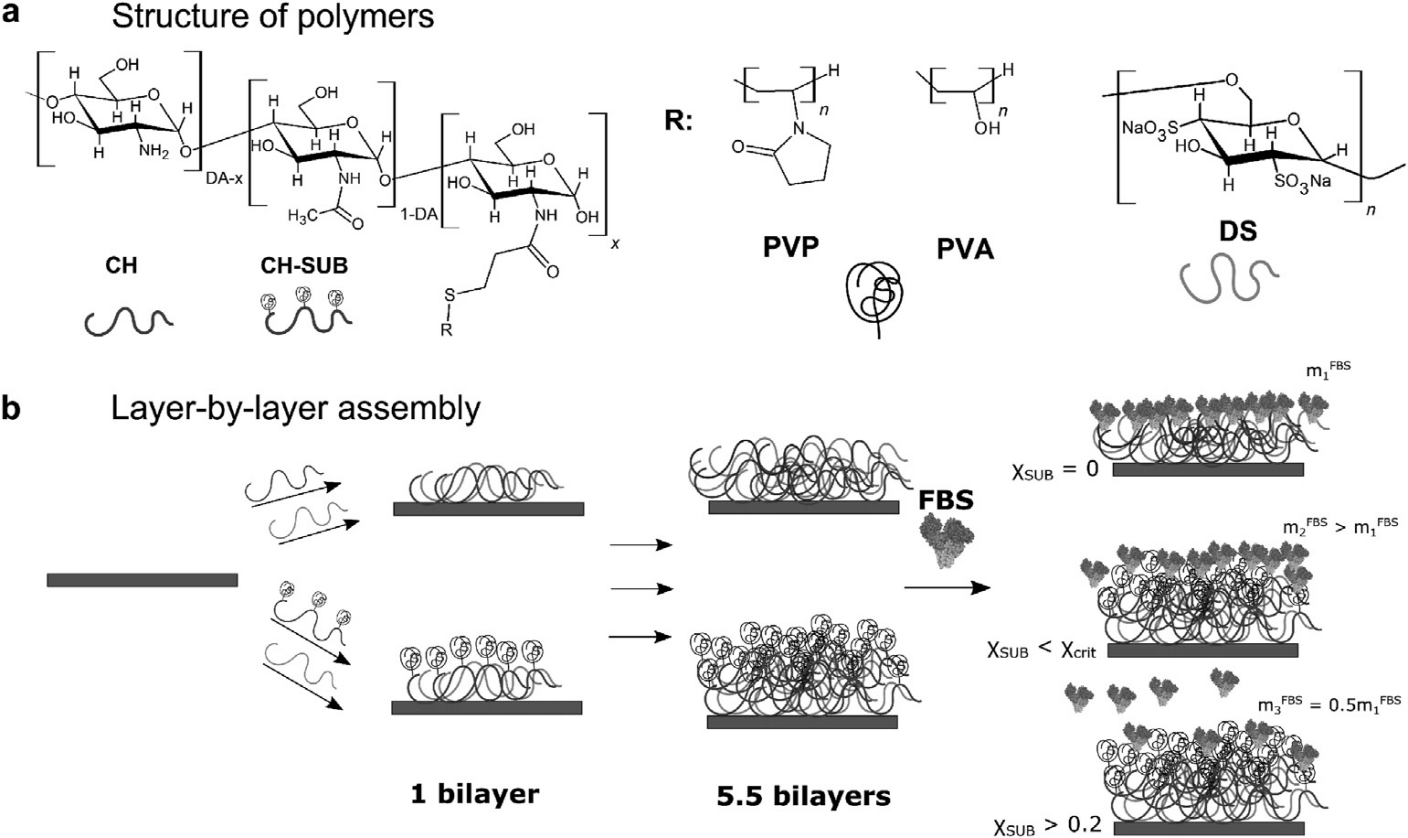
Schematic representation of polymers' structure (a) and the LbL assembly process of (CH-SUB/DS)_n_ films and FBS protein repellency (b).

In this work we report on the preparation of LbL films based on graft copolymers of chitosan with PVP or PVA and dextran sulfate and characterization of their thickness, hydrophilicity, surface roughness, and protein repellency (Figure 1). The synthetic sequence for PVP and PVA of low molecular weight (2.4 and 2.0 kDa, respectively) with one carboxylic end group that allows us to utilize carbodiimide crosslinking to obtain copolymer of chitosan with controllable degree of amine group substitution is proposed.

## 2 Materials and methods

### 2.1 Materials

Chitosan of medium molecular weight (CH, ca. 450 kDa, Sigma, deacetylation degree (DA) 0.75), dextran sulfate (DS, >500 kDa, Sigma), polyethyleneimine (PEI, 250 kDa, 50 % w/v solution, Sigma), polystyrene sulfonate (PSS, 70 kDa, Sigma), N-vinylpyrrolidone (VP, NaOH stabilized, Sigma), vinyl acetate (VAc, hydroquinone stabilized, Sigma), 4,4′-azobis(4-cyanovaleric acid) (ABVA), mercaptopropionic acid (MPA, Merck), 1-ethyl-3-(3-dimethyl aminopropyl) carbodiimide (EDC), fluorescein isothiocyanate (FITC, Sigma), morpholinoethyl sulfonic acid (MES, Roth), fetal bovine serum (FBS, South America origin, BioClot GmbH) were used as received without additional purification.

### 2.2 Synthesis and characterization of hydrophilic oligomers

**Figure 2:**
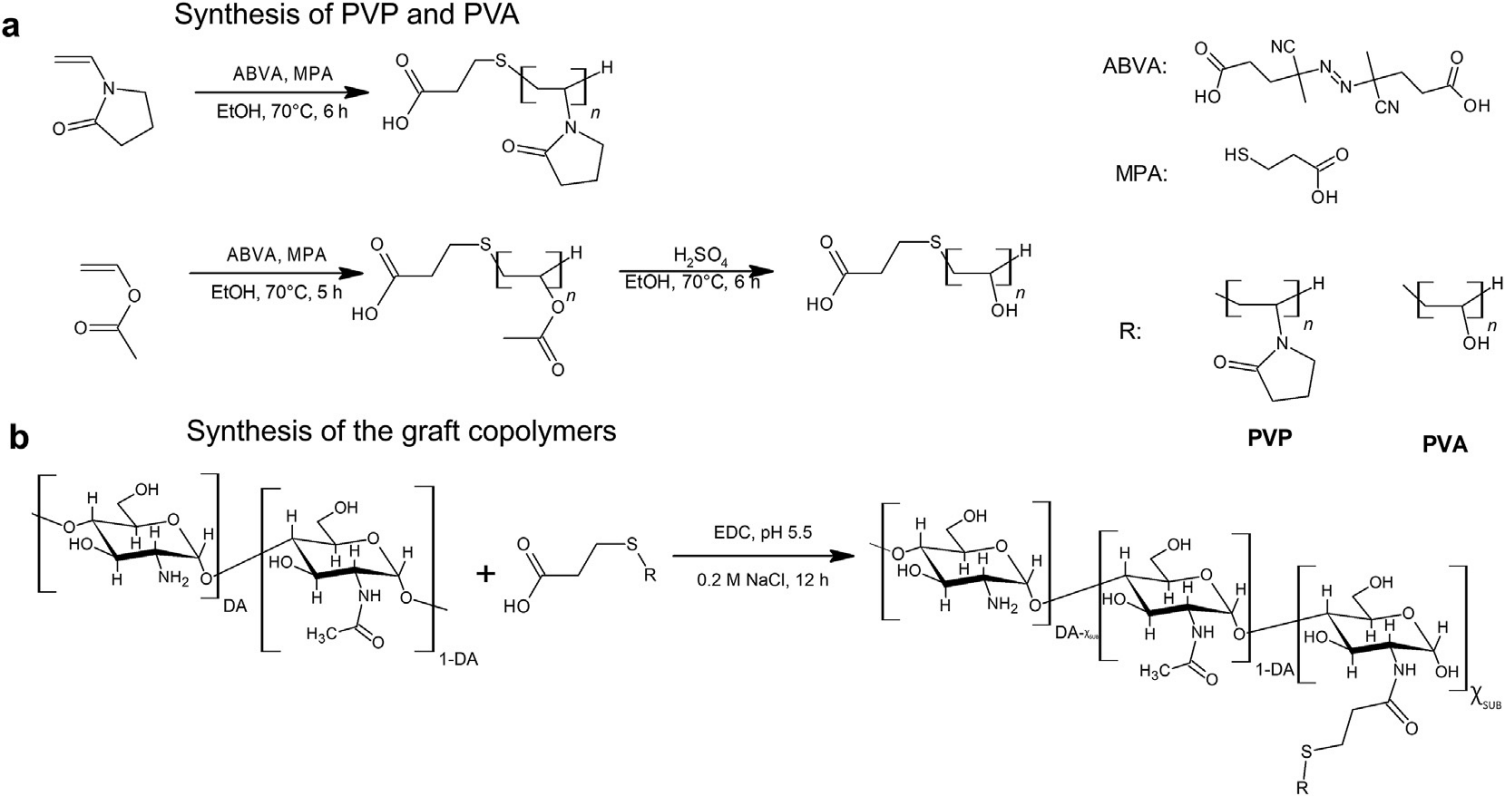
Scheme of synthesis of low molecular weight PVP and PVA with carboxylic end group (a) followed by their grafting to chitosan backbone (b).

Poly(N-vinylpyrrolidone) (PVP) and poly(vinyl acetate) (PVAc) of low molecular weight with one carboxylic end group were obtained by radical polymerization of suitable monomers using mercaptopropionic acid as a chain transfer agent (Figure 2). The detailed protocols are given in S1 and S2 sections in the Supplementary file. The application of MPA allows one to finish the preceding polymerization chain by subtracting hydrogen atom from a weak mercapto group of MPA and initiate the next with the radical containing carboxylic group. The addition of MPA into the reaction mixture with a constant rate controls the concentration ratio of monomer to MPA. By varying this ratio, the number molecular weight (M_n_) of the obtained oligomeric PVP and PVAc is controlled [29, 30]. Poly(vinyl alcohol) (PVA) was obtained by acid hydrolysis of PVAc of appropriated M_n_.

M_n_ of PVP and PVA calculated from carboxylic groups titration of the polymers with a potassium hydroxide solution using phenolphthalein as an indicator are 2.4 and 2.0 kDa, respectively. The hydrolysis degree of PVA was calculated using FTIR-spectroscopy (Figure S1 in the Supplementary file). The ratio of absorption peaks at 1750 cm^−1^ (C=O bond, stretching) and 1450 cm^−1^ (C–H bond, scissoring) was 1.05 and corresponds to the degree of hydrolysis of PVA equal to 80–85 % [31].

Grafted copolymers of chitosan with side chains of hydrophilic vinyl oligomers (CH-SUB, where SUB = PVP or PVA) were synthesized using crosslinking agent EDC (Figure 2). Briefly, chitosan and vinyl oligomers were mixed in a suitable ratio (Table S1 and S2 in the Supplementary file) of non-ionogenic polymer chains to glucosamine elemental units (section S4 in the Supplementary file). The mixture was treated with EDC in 0.1 M MES buffer supplemented with 0.2 M sodium chloride (pH 5.5 ± 0.2) for 12 h, dialyzed successively against 0.2 M NaCl and water (a Sigma skin-tube membrane, cutoff 12.4 kDa). To obtain CH with solubility and counterion concentration comparable to that of copolymers, chitosan was dissolved in 0.02 M HCl and dialyzed in the same manner as CH-SUB [32]. The degree of substitution (χ_SUB_), or fraction of –NH_2_ group bonded to SUB, was determined spectrophotometrically as described elsewhere [33].

### 2.3 Preparation and characterization of LbL films

LbL films consisting of a given number of CH-SUB/DS bilayers were formed on various substrates using 1 mg/mL polyelectrolyte solutions in distilled water at room temperature. All solutions were prefiltered through a 0.46 μm syringe filter (RC material, Agilent) directly before use. To dissolve copolymers of chitosan with PVA, the solutions were heated to 70–80 °C for 10 min under mixing. All substrates were preliminary hydrophilized in the mixture of H_2_O_2_:NH_3_:H_2_O (1:1:3 by volume), rinsed with water, and dried in the argon stream.

### 2.4 Atomic force microscopy (AFM)

Silicon (110) substrates were premodified with 3 bilayers of PEI/PSS and the assembly of 5.5 bilayers of CH-SUB/DS was carried out on the surface. The substrates were dipped into a polyelectrolyte solution for 5 min followed by rinsing twice with distilled water after each adsorption step. The obtained LbL films were dried in argon stream and stored at ambient temperature and humidity for 24 h before the measurement.

An *Certus, Nanoflex* atomic force microscope with an n-type silicon cantilever (8 nm tip) in the tapping mode was used to determine the surface morphology of LbL films. The scan area was 2.5 × 2.5 μm. The root mean square height (S_q_) was calculated using the Gwyddion software. Scanning was performed on three different areas for each film and the obtained values were averaged.

### 2.5 Static contact angle measurement

Glass slides were modified with a precursory (PEI/PSS)_3_ film. Then, 1 or 5.5 bilayers of CH-SUB/DS were assembled on the surface according to the same protocol as described above. After formation of required number of bilayers, slides were dried in argon stream, stored for 2 h under ambient conditions and the measurement of contact angle was performed. A drop of water (2.5 ± 0.5 μL) was placed on the surface of planar film and equilibrated for 30 s. A photograph of the drop was handled in the ImageJ software equipped with LB-ADSA drop analysis plugin [34]. The value of static contact angle (θ) was averaged for 8 drops placed on 2 different substrates (4 drops on each substrate).

### 2.6 Quartz crystal microbalance (QCM) study

A *QCM200, Stanford Research Systems* instrument that measures frequency (F) and motional resistance (R) of a resonator placed in a flow cell was used to study the properties of LbL films based on chitosan or its copolymers and dextran sulfate. Quartz sensors (Au-coated, AT-cut, nominal frequency 5 MHz) were used as templates for the assembly of LbL films. The deposition of LbL films and adsorption of FBS were performed according to the previously reported protocol with minimal modification [28].

The adsorbed mass (*m*_*w*_) in water was calculated using the Sauerbrey equation [35]:

(1) mw=−ΔFC

where ΔF is the change of frequency of a quartz resonator after each adsorption (including washing) step, Hz; C is the device constant (56.6 Hz μg^-^^1^ cm^2^). The film thickness was calculated taking the film density equal to 1.05 g/cm^3^ [36].

### 2.7 Determination of dry polymer adsorbed mass and water content in the LbL films

The mass of CH-SUB per cm^2^ (m_CH-SUB_) in the LbL films was evaluated using copolymers labeled with FITC. The protocol of copolymer labeling is described elsewhere [28].

The LbL films were formed by dipping glass slides in 1 mg/mL solutions of FITC-labeled CH-SUB and DS with intermediate washings in DI water. The absorption spectra of the films in the visible range were recorded using a *CM 2203, Solar* spectrofluorometer. The mass of CH-SUB per cm^2^ (m_CH-SUB_) in a film was recalculated from the absorbance of the film at 496 nm in PBS buffer using a 1:1 (w/w) mixture of FITC-labeled copolymer and DS for calibration.

The mass of dry matter per cm^2^ in an LbL film (m_*d*_) was determined as

(2) $$m_{d}=\frac{m_{CH-SUB}}{w_{CH-SUB}}$$

where *w*_CH-SUB_ is the mass fraction of copolymer in the film calculated assuming electroneutrality of the LbL film material and the presence of 2.8 negative charges per one DS structural unit [37].

The water content in an LbL film was evaluated as

(3) $$w_{H_{2}O}=\frac{m_{w}-m_{d}}{m_{w}}\cdot 100\%$$

The amount of PVP or PVA chains per cm^2^ of LbL film was calculated as

(4) $$n_{SUB}=\frac{m_{CH-SUB}\cdot v_{SUB}}{M_{SUB}}$$

where *v*_SUB_ is the mass fraction of SUB in copolymer calculated on the basis of its deacetylation degree and χ_SUB_, *M*_*SUB*_ is the molecular weight of PVP or PVA.

The average spacing between the SUB chains (*L*) was calculated assuming a hexagonal (close-packed) arrangement of SUB on the surface [38]:

(5) $$L={\left(\frac{2}{\sqrt{3}n_{SUB}N_{a}}\right)}^{0.5}$$

where N_a_ is Avogadro's constant.

## 3 Results and discussion

### 3.1 CH-SUB copolymers

Chitosan is covalently modified with side chains of PVP 2.4 kDa or PVA 2.0 kDa and graft-copolymers CH-SUB with various degree of amine group substitution by the short polyvinyl side chains are obtained. The value of χ_SUB_ is precisely controlled by the ratio of the quantities of carboxy-terminated PVP or PVA chains to deacetylated glucosamine units in the reaction mixture. According to the assay of primary amino groups [33] χ_SUB_ in CH-SUB is in the range of 0.01–0.32 mol/mol. Despite this and the fact that the molar fraction of N-acetylated glucosamine groups in CH is 0.25 ± 0.03 mol/mol, the backbone of synthetized copolymers CH-SUB contains at least 43 % of glucosamine groups that are responsible for positive electrostatic charge of the macromolecule and LbL interaction with negatively charged dextran sulfate.

### 3.2 Physicochemical properties of (CH-SUB/DS)_n_ films

**Figure 3:**
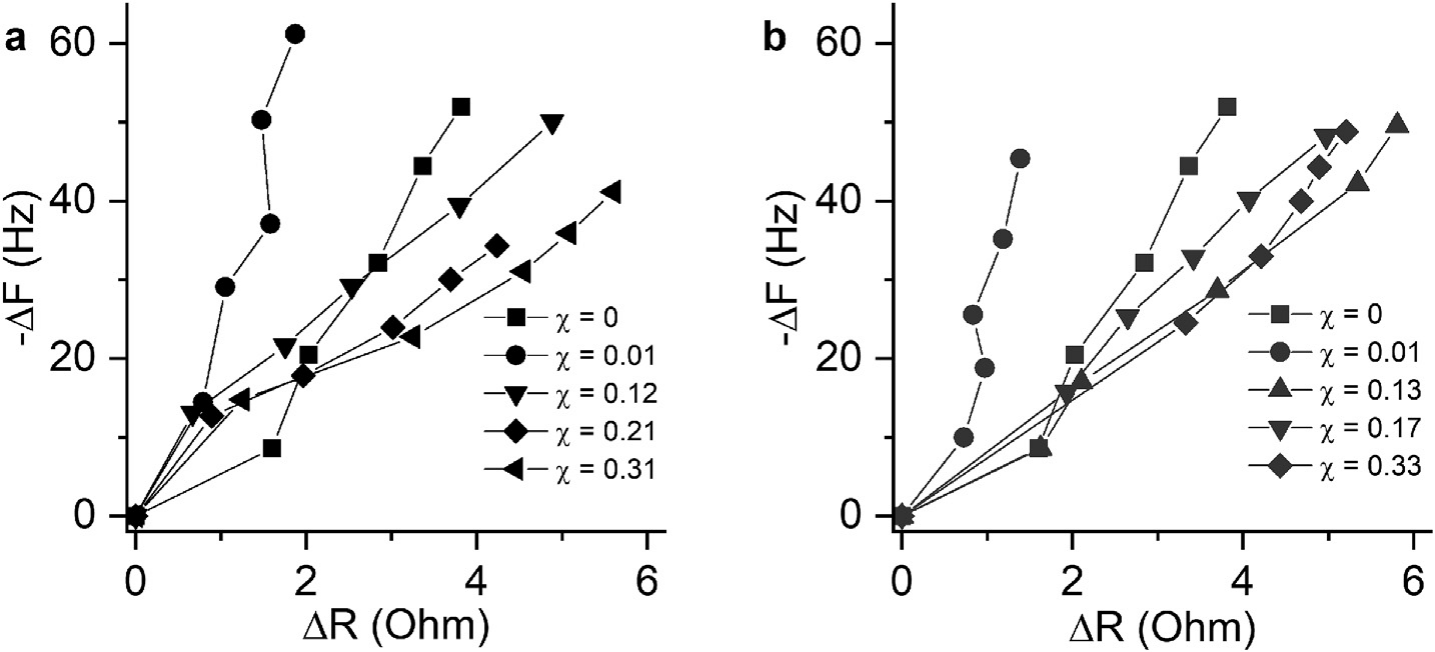
Changes in frequency and motional resistance during formation of (CH-PVP/DS)_n_ (a) and (CH-PVA/DS)_n_ (b) films. Each point corresponds to deposition of a CH-SUB/DS bilayer. The values are averaged from three independent measurements.

The typical frequency changes of a 5 MHz resonator in the process of layer-by-layer assembly of CH-SUB and DS on its surface are shown in Figure S2 in the Supplementary file. Figure 3 represents -ΔF and ΔR obtained upon adsorption of each CH-SUD/DS bilayer in timeless coordinates. The formation of a (CH-SUB/DS)_n_ film is accompanied by a sharp decrease of resonant frequency and a moderate change of motional resistance. The changes escalate with increasing the number of bilayers in a film. Motional resistance characterizes acoustic dampening of a deposit on the surface of quartz resonator [39], so the ratio of ΔF to ΔR values can be used to compare the viscoelastic properties of a film assembled on its surface [40].

For both CH-SUB copolymers at χ_SUB_ ≥0.12, acoustic dampening of the LbL films, which is qualitatively determined by the -ΔF/ΔR ratio, is comparable or slightly less to that for (CH/DS)_n_ films. On the contrary, the formation of LbL films of copolymers with low degree of substitution (χ_SUB_ = 0.01 ​mol/mol) is accompanied by significant increase in - ​ΔF/ΔR value due to low ΔR per bilayer (Figure 3).

**Figure 4:**
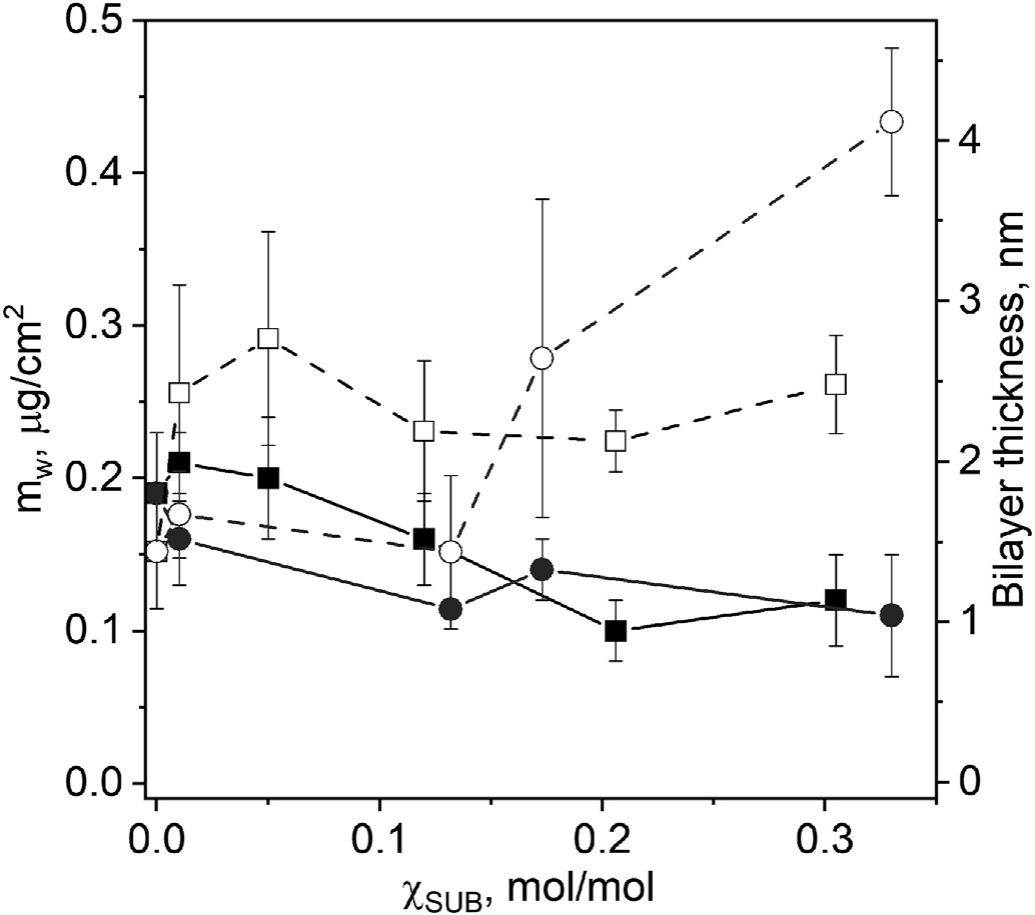
Masses and thicknesses of the first (open symbols) and the following (solid symbols) (CH-SUB/DS) bilayers as a function of χ_SUB_. SUB: PVP (squares) or PVA (circles).

A separate analysis of the wet masses and thicknesses (Eq. 1) of the first and the subsequent layers (Figure 4) provides a valuable information on the structure of (CH-SUB/DS)_n_ films and features of the assembly process. Being independent of χ_SUB_, the thickness of the first CH-PVP/DS bilayer is ∼1.0 nm higher than that of the first CH/DS bilayer (2.4 ± 0.3 and 1.4 ± 0.5 nm, respectively). In contrast, the thickness of the first CH-PVA/DS bilayer has the same value as unmodified chitosan-based bilayer at χ_SUB_ lower than 0.1 mol/mol and significantly increases only at χ_SUB_>0.12 mol/mol reaching 4.0 nm at the highest investigated degree of substitution. The observed peculiarities apparently related with the difference in sizes of PVP and PVA side chain coils in water.

Unfortunately, most reported values of radius of gyration R_g_ or hydrodynamic radius (R_h_) of PVP and PVA coils estimated by a suitable experimental method in good solvent were obtained for high molecular weight polymers [41, 42, 43, 44, 45, 46, 47]. For example, the radius of gyration in methanol for PVP of 6–574 kDa is given by the equation R_g_ = 0.0195⋅M^0.55^ found from the intrinsic viscosity dependence on polymer molecular weight [41]. For PVP 10–360 kDa, R_h_ = 0.0241⋅M^0.575^ in methanol [42] and R_h_ = 0.0458⋅M^0.521^ in water [43] are proposed from DLS measurements at 25 °C; while R_h_ = 0.0136⋅M^0.583^ can be obtained by mathematically processing the results in [44]. By photon correlation spectroscopy, R_h_ of several commercially produced PVP of 10.5–1,150.0 kDa is found to be in the range of 2.75–34.5 nm (R_h_ = 0.036⋅M^0.499^) with a R_h_/R_g_ ratio equal to 0.72–0.84 [45]. Calculated from mentioned above dependences extrapolated to PVP 2.4 kDa the value of R_h_ varies 1.27–2.64 nm, and, therefore, R_g_ is from 1.4 to 4.0 nm.

The only reported value of hydrodynamic radius of low molecular weight PVP (2.5 kDa) was found from quasi-elastic light scattering and intrinsic viscosity and it is ca. 1.23 nm [48]. An estimation of R_g_ from the mentioned R_h_ and mean R_h_/R_g_ ratio of 0.78 is close to 1.58 nm. Since the molecular weight of PVP side chains in CH-PVP copolymers is only slightly lower (2.4 kDa), the values of R_h_ and R_g_ were used as an estimation of PVP coil dimensions. The thickness of the first CH-PVP/DS bilayer (∼2.4–2.5 nm) is consistent with the double value of R_h_ and slightly smaller than 2R_g_. Apparently, upon adsorption of the first bilayer PVP side chains are situated on the surface of polyelectrolyte part of the bilayer in the vicinity of copolymer backbone. Indeed, it is known that PVP itself adsorbs on a variety of materials through hydrogen bonding [49] and can be slightly flatten on their surface [50]. Moreover, with an increase in χ_SUB_, there are no changes in the mode of the first bilayer formation.

Similarly, the dimensions of high molecular weight PVA coils are well studied. For PVA 115.5 kDa, R_g_ = 15.8 ± 0.7 nm was found by small-angle neutron scattering measurements. For ∼47 kDa Mowiol 6–98, effective radius (R_eff_) in the range of 5.7–8.5 nm was calculated from viscosity of diluted solutions of DMSO and water at 293–353 K [46]. For PVA with a degree of polymerization from 500 (∼24 kDa) to 8000, R_h_ = 6–26 nm with R_g_/R_h_ = ∼1.53–1.58 was evaluated by intrinsic viscosity and laser light scattering [47]. For PVA containing 30 repeat units (N) in trans-configuration, the radius of gyration of 0.7–1.1 nm and the end-to-end distance of 1.2–1.8 (293–323 K) were calculated using GROMAC software [51]. Molecular dynamic calculations for melts of PVA oligomers with 1–10 repeat units are summed into the formula: R_g_ = 0.135⋅N^0.65^^±^^0.03^ [52]. The extrapolation of the later equation to 44 units as in PVA 2.0 kDa gives R_g_ = 1.58 nm.

Taking into account the dimensions of PVA 2.0 kDa coils it is reasonable to assume that at small χ_SUB_, PVA chains in the first CH-PVA/DS bilayer are situated below the average level of polyelectrolyte loops and flatten on the surface (“pancake” conformation) [14]. With increasing chain concentration on the interface neighboring PVA side chains apparently associate [53] and are pushed above the surface, thus increasing the apparent bilayer thickness.

Calculated mass of a (CH-SUB/DS)_n_ film and, therefore, its thickness between the fifth and first bilayer increases linearly with n. It allows us to calculate the average bilayer thickness. The thickness of a CH-SUB/DS bilayer tends to decrease slightly with increasing χ_SUB_ (Figure 4), from 1.5 nm for CH/DS to approximately 1.1 nm for CH-PVP/DS or CH-PVA/DS with χ_SUB_ ∼0.3 mol/mol. Regardless of SUB, the bilayer increment of film growth is lower than the thickness of the first bilayer. А possible explanation involves adsorption of poly(N-glucosamine) part of CH-SUB on oppositely charged surface in the gaps between SUB of previously adsorbed layers, thus adding new SUB chains and stepwise forming a dense SUB layer on the surface of a LbL film. The lower bilayer thickness (adsorbed mass) at higher value of χ_SUB_ fits well with the proposed mechanism.

### 3.3 Water content in LbL films

**Figure 5:**
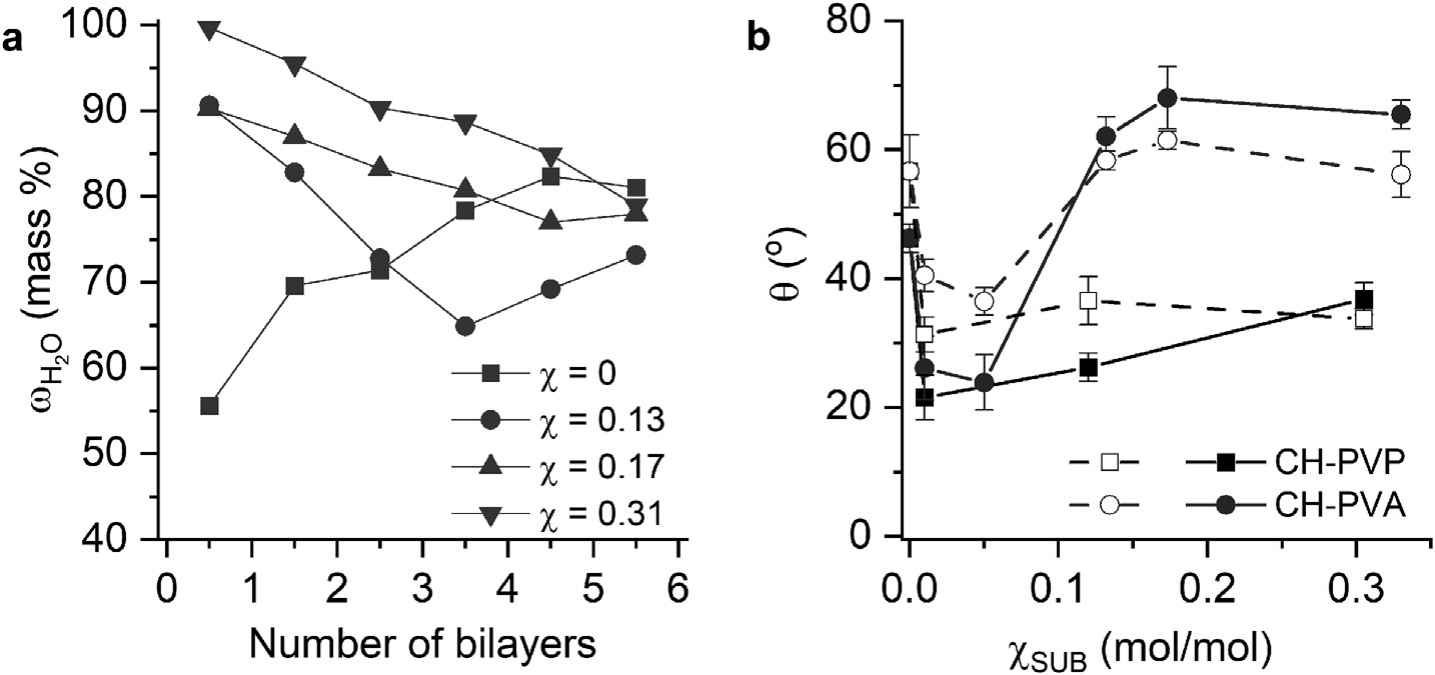
a) Water content in a (CH-PVA/DS)_n_ film vs. number of bilayers. b) Dependence of contact angle (θ) on χ_SUB_ for (CH-SUB/DS)_n_ films with n = 1 (open symbols, dashed lines) or 5.5 (solid symbols and lines).

Calculated from absorbance at 496 nm (A_496_) dry mass of the (CH-PVA/DS)_n_ films obtained using FITC-labeled copolymers with different χ_SUB_ (Eq. 2) increases linearly with the number of bilayers (Figure S3 in the Supplementary file). Figure 5a shows the mass percentage of water in a (CH-PVA/DS)_n_ film as estimated from wet, obtained by QCM, and dry, calculated from absorbance assuming electroneutrality of the material, masses of the film (Eq. 3).

If copolymer with a high degree of substitution is applied the water content in a 0.5–2.5 bilayer CH-PVA/DS film increases above 80% vs. 55–70 % for unmodified chitosan. Similar to the LbL films on PEG and DEX copolymers [28], a reciprocal dependency of water mass percent on the number of bilayers is observed due to displacement of water from internal layers [54]. For the films consisting of 3.5–5.5 CH-PVA/DS bilayers, the percent of water decreases to the value for the films of unmodified chitosan, for which the water content increases with n.

Unfortunately, the presence of PVP strongly interferes with spectral characteristics of most common xanthene dyes (fluorescein, eosin) and benzidine-based azo-dyes (Congo red) [55, 56, 57]. A decrease of A_496_ of a 1 μg/mL FITC solution was experimentally found as the concentration of PVP increases (Figure S4 in the Supplementary file). It means that no proportionality is expected between the surface concentration of PVP and optical properties of the LbL films based on FITC-labeled copolymers. Furthermore, analytical assays for PVP do not show the desired sensitivity, while HPLC is impracticable for the insoluble films. Consequently, the evaluation of water content and side chain overlapping parameter for the (CH-PVP/DS)_n_ films does not seem possible.

### 3.4 Wettability of (CH-SUB/DS)_n_ films

The wettability of (CH-SUB/DS)_n_ films depends on χ_SUB_ (Figure 5b). Unmodified chitosan under good solvent condition forms with DS relatively hydrophobic surface with a contact angle of 56±3°. This value is comparable with the wettability of the precursor 3 bilayer film assembled from PEI and PSS (62±2°). Introducing the minimal number of PVP chains into (CH-PVP/DS)_n_ films by using copolymer with χ_SUB_ = 0.01 mol/mol significantly decreases the contact angle value. Staying within the range typical for PVP highly grafted surfaces [58] the value of contact angle increases slightly with the further growth of χ_SUB_.

Similar to CH-PVP containing films, those based on CH-PVA also demonstrate a decrease of contact angle at low substitution degree (<0.05 mol/mol). At the same time, the inclusion of CH-PVA with a high χ_SUB_ leads to significant surface hydrophobization. The contact angle increases to a value as high as that of the precursor film. The obtained values (55-65°) are comparable with previously reported contact angles of PVA physisorbed films and PVA-chitosan (40:1) blends (55-65°) [53, 59]. This behavior can be attributed to association of closely packed PVA side chains through strong intra- and intermolecular hydrogen bonding [60, 61] upon film drying and formation of dry PVA overcasting layer. Thus, there is no contradiction between the observed contact angle of dry LbL films and high water content in the wet ones.

As seen from Figure 5b, for the films consisting of one CH-SUB/DS bilayer, the dependence of the contact angle on χ_SUB_ differs from the thicker films only in magnitude. It means that the structure that determines the hydrophilic properties of the copolymer-based films is formed upon adsorption of the first bilayer.

### 3.5 Surface morphology of (CH-SUB/DS)_n_ films

**Figure 6:**
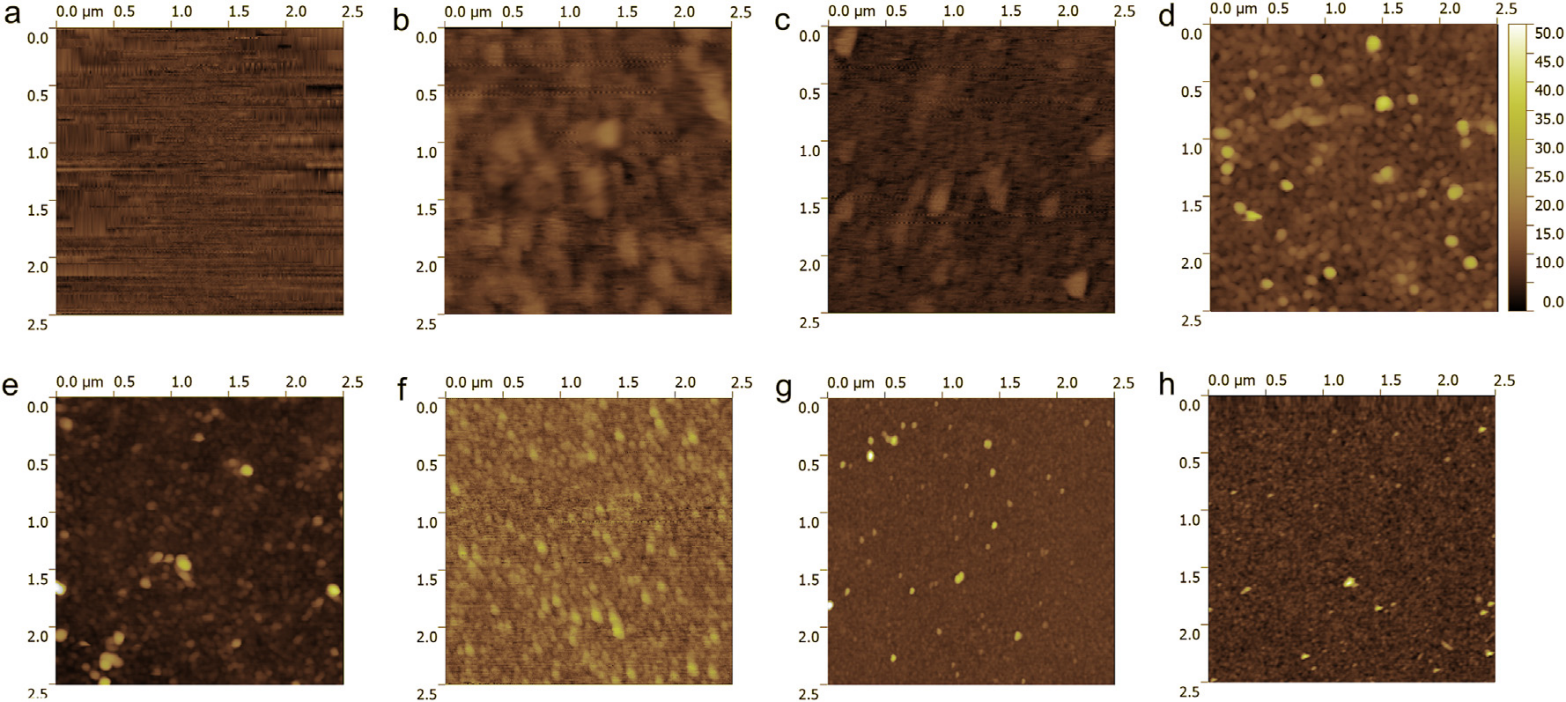
AFM micrographs of surfaces of a precursor (PEI/PSS)_3_ (a), (CH/DS)_5.5_ (e), and (CH-SUB/DS)_5.5_ films. SUB: PVP (b-d, χ_SUB_ (mol/mol): 0.01 (b), 0.12 (c), 0.31 (d)); PVA (f-h, χ_SUB_ (mol/mol): 0.01 (f), 0.13 (g), 0.33 (h).

AFM images of the surface of a precursor (PEI/PSS)_3_ film and (CH-SUB/DS)_5.5_ films assembled on the basis of CH-SUB with different SUB and χ_SUB_ are shown in Figure 6. Despite being continuous and uniform, all chitosan and copolymer–based assemblies are characterized by the presence of grains protruding to a height of less than 50 nm above the average film level (Figure 6).

**Figure 7:**
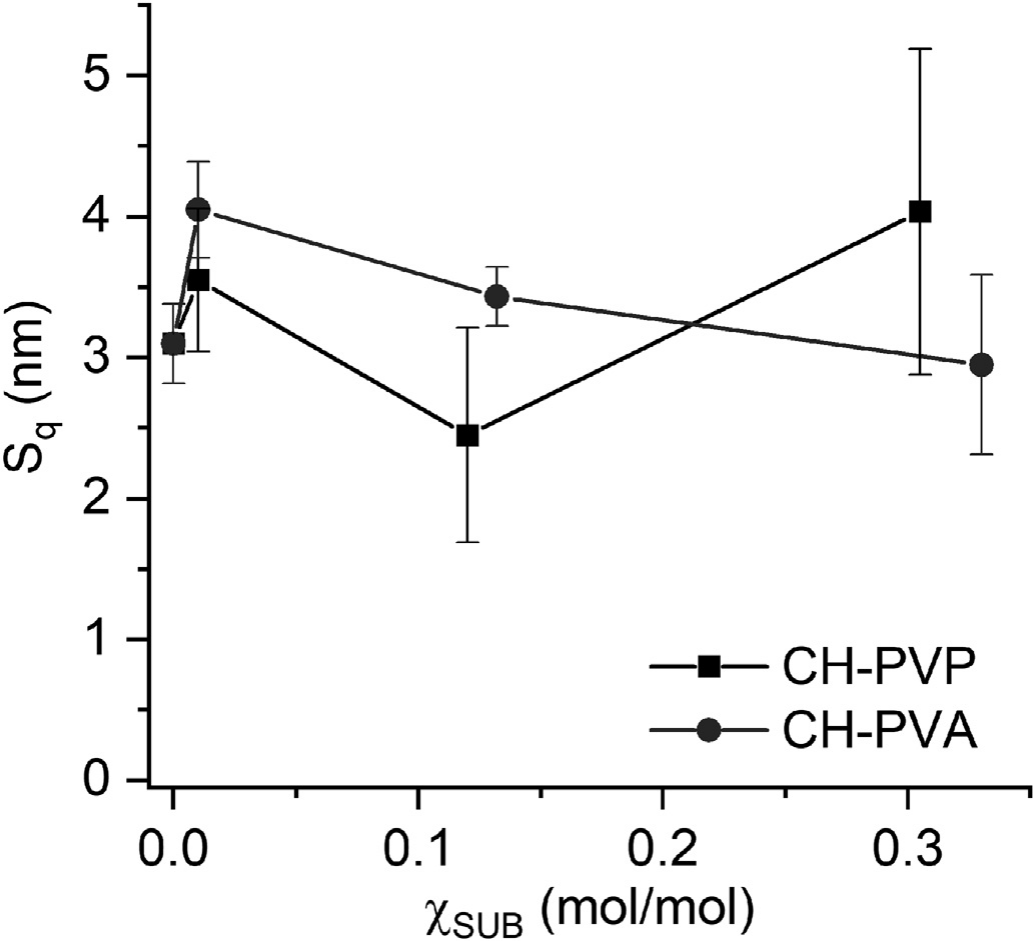
Surface roughness of (CH-SUB/DS)_5.5_ films vs. degree of amine group substitution by SUB.

The calculated surface roughness of (CH/DS)_5.5_ films is 3.1 ± 0.3 ​nm (Figure 7). The replacement of CH by CH-SUB, where SUB = PVP or PVA, is accompanied by morphological changes which are specific for copolymer-based LbL films. For both copolymers, there is an increase of surface roughness at low χ_SUB_ (∼0.01 mol/mol) followed by a small decrease at medium substitution degree (∼0.1–0.15 mol/mol). The similar changes of surface morphology were previously observed by AFM for LbL films on the basis of copolymers of chitosan with polyethylene glycol and dextran [28]. For copolymers with χ_SUB_ ∼ 0.3 mol/mol, the difference between side chains becomes more pronounced. For CH-PVP, S_q_ almost doubles as compared with the value at a medium χ_SUB_ due to increased number of grains with a diameter of 200–500 nm. On the contrary, the introduction of additional PVA side chains in copolymer leads to further decrease of roughness of the LbL films. These changes of surface morphology are apparently caused by a combination of several interfacial phenomena occurring upon film drying, such as phase separation prevailing in the case of copolymers with PVP and conformational changes developing in the case of CH-PVA macromolecules [62].

### 3.6 Adsorption of FBS proteins

Protein resistant properties of LbL films on the basis of copolymers CH-SUB were investigated using fetal bovine serum as a source of proteins (albumin and globulins) that are major components of FBS [63]. Adsorption of proteins is the first stage of interaction of serum with any surface, including that of a LbL film [64].

The wet mass of FBS proteins (m_FBS_) adsorbed on the surface of a (CH-SUB/DS)_5.5_ film was calculated from the drop of frequency (ΔF_FBS_) of a resonator with the film after a 60 min exposure to FBS and subsequent washing with water (Figure S2) using Eq. (1). As the Sauerbrey equation doesn't take into account the viscous load of adsorbate, it slightly overestimate the wet mass of adsorbed protein [65]. Nevertheless, the divergency with the viscoelastic model does not exceed 10 % for thin protein layers [66]. The changes of motional resistance after adsorption of FBS proteins (ΔR_FBS_) on the (CH-SUB/DS)_n_ films are small (∼7 Ohm), and the impact of the viscous load of the protein layer was ignored. As the same experimental conditions are used for all the copolymer based LbL films their protein resistant properties are compared on the basis of m_FBS_.

**Figure 8:**
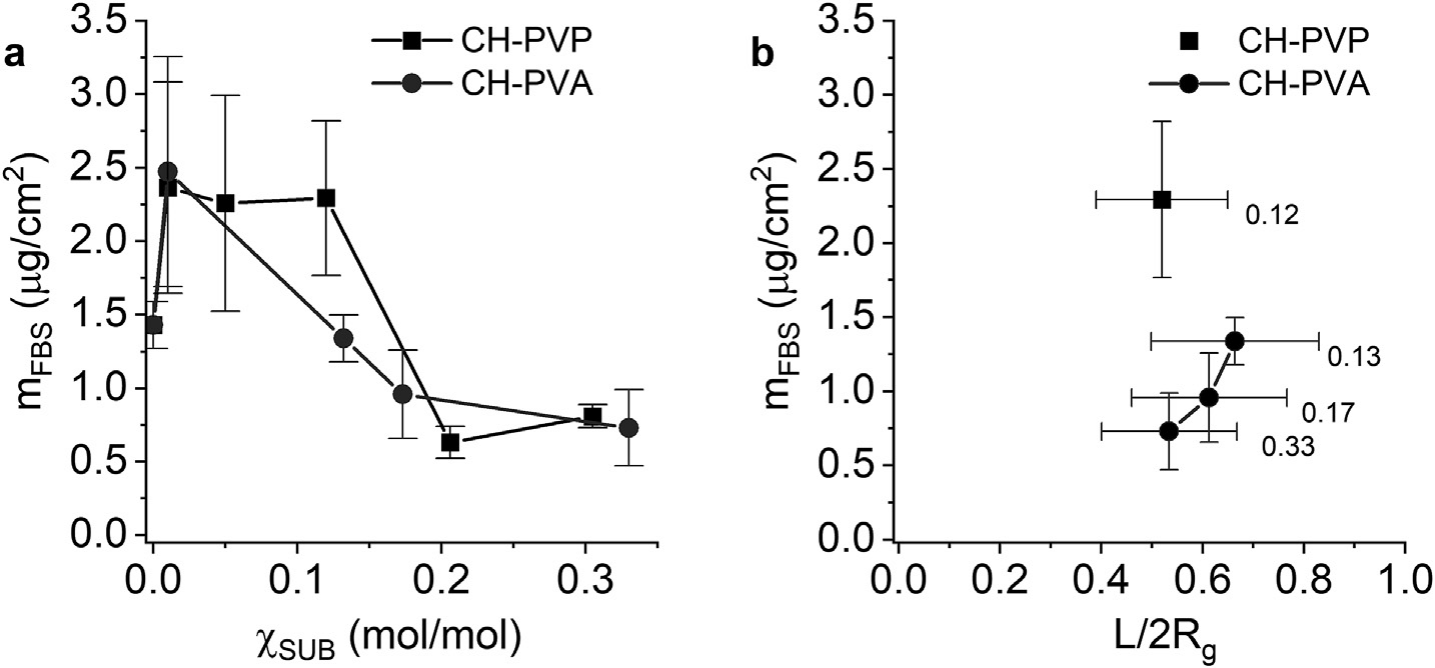
Mass of FBS proteins adsorbed on the surface of (CH-SUB/DS)_5.5_ films as a function of χ_SUB_ (a) and L/2R_g_ (b, the values of χ_SUB_ are shown next to experimental points).

A CH-based LbL film readily adsorbs FBS proteins on its surface (Figure 8a). The introduction of SUB onto CH backbone changes the protein resistance of the LbL films; m_FBS_ depends on the degree of substitution and the nature of grafted chains. The LbL films on PVP-based copolymers do not show any protein repellent properties at χ_SUB_ lower or equal to 0.12 (χ_crit_). On the contrary, the small number of PVP side chains in the LbL film promotes protein adhesion. Only at χ_SUB_ as high as 0.2 mol/mol the mass of FBS proteins adsorbed on a (CH-PVP/DS)_5.5_ film drastically decreases. Similar but less persevering increase of protein adsorption was found for (CH-PVA/DS)_5.5_ films at the lowest investigated χ_SUB_ = 0.01 mol/mol, and as more PVA chains cover the surface m_FBS_ inclines.

One of possible explanations of the FBS protein adsorption trends includes analysis of the morphological characteristics of the LbL surfaces. For both CH-PVP and CH-PVA based films, an increase of roughness at low χ_SUB_ is observed (Figure 7). Under this assumption, steadily decreasing protein adsorption on (CH-PVA/DS)_5.5_ films and their S_q_ with increasing χ_SUB_ are in good agreement.

From another point of view, the results on thickness of adsorbed CH-SUB/DS bilayers (Figure 4) suggest that, at low χ_SUB_, PVP and PVA coils strongly interact with underlying charged layers, and this can distort their shape and expose their hydrophobic moieties to proteins.

Apparently, the adsorbed PVP chains have a more “coil-like” structure, while the PVA conformation rather resembles a “pancake”. In this case, the adsorption of proteins on each of CH-SUB can follow a different path. Protein repellency of PVP-grafted surfaces depends on the average interchain distance and no resistance of modified surface to protein adsorption is observed until the distance between PVP chains is larger or comparable with protein size [67]. Contrariwise, chemisorbed PVP macromolecules can enhance BSA adsorption [15, 16], as we observed experimentally. The study [68] on the adsorption of Pluronic F-108 (block copolymer of hydrophilic ethylene oxide and hydrophobic propylene oxide monomers) suggests that on a hydrophilic substrate such copolymer is adsorbed in the “pancake” conformation. Despite the mass of subsequently adsorbed lipase protein decreased with an increase in the number of Pluronic F-108 chains, the adsorption was not completely blocked by the polymer layer due to the presence of hydrophobic regions. The PVA side chain molecules can also be modeled after a copolymer consisting of hydrophilic hydroxyvinyl and hydrophobic vinyl acetate moieties (85 and 15%, respectively, as found by FTIR). Probably, interfacial behavior of the PVA chains is similar to that of F-108. An increase of PVP and PVA surface concentration at higher degrees of substitutions leads to coils overlapping, on the one hand, and the conformational changes from “pancake” to elongated coil or even brush (quasi-brush), on the other [69, 70]. Thus, the effect of flattened side chains on protein adsorption weakens at high χ_SUB_.

A comparison of m_FBS_ on the surface of a 5.5 bilayer film based on CH and CH-SUB shows that a 50 % reduction of proteins adsorption is reached if the used CH-PVP and CH-PVA have χ_SUB_ above 0.2. If the mentioned value is compared with corresponding characteristics for PEG and DEX-based copolymers of chitosan (0.015 and 0.1 mol/mol, respectively) [28], the PVP and PVA copolymers seem to be less effective in suppression the protein adsorption. However, the molecular weights of the previously used PEG and DEX side chain polymers were 2–3 times large (5.0 and 6.0 kDa, respectively) than M_n_ of PVP and PVA used in this study (2.4 and 2.0 kDa, respectively). This leads to a greater distance between SUB chains on the film surface at comparable value of χ_SUB_. In the range of χ_SUB_ from 0.2 to 0.3 the bioinert properties of multilayers based on the copolymers with different SUB become comparable for all (CH-SUB/DS)_5.5_ films, including PEG and DEX based copolymers.

### 3.7 Correlation between FBS adsorption and overlapping parameter *L*/2*R*_*g*_

Protein repellency of polymer surfaces grafted with non-ionogenic hydrophilic chains depends on the average interchain distance *L* (Eqs. (4) and (5)). If the distance is larger or comparable to protein size, the resistance of modified surface to protein adsorption is not observed [26, 38, 67].

The dependence of mass of the adsorbed proteins on the overlapping parameter *L/2R*_*g*_ for (CH-PVA/DS)_5.5_ films is shown in Figure 8b. As an estimate of PVA 2.0 kDa coil radius on the surface of the films, a value of 1.6 nm [52] was used.

Similar to other copolymer-based films, the noticeable decrease of protein adsorption on (CH-PVA/DS)_n_ takes place if the overlapping parameter is getting smaller than 1.0. The value matches the overlapping grafted side chains. The minimum adsorption of proteins on (CH-PVA/DS)_5.5_ films is achieved at χ_SUB_ = 0.3 mol/mol where the *L*/2*R*_*g*_ value is ca. 0.55. This is in good agreement with the previously obtained results for the films on PEG and DEX–based copolymers. *L*/2*R*_*g*_ lower than 0.5 is required to inhibit adsorption of FBS proteins on (CH-PEG/DS)_n_ and (CH-DEX/DS)_n_ films of similar structure [28].

Unfortunately, due to strong influence of PVP concentration on FITC absorption, the evaluation of *L* for (CH-PVP/DS)_n_ films is difficult and reliable results are obtained only for the films based on CH-PVP with the low degree of substitution (0.12 mol/mol) and calculated R_*g*_ = 1.6 ​nm [48] as shown in Figure 8b. Other methods due to analytes interaction with polyelectrolyte part of the films and insufficient sensitivity were proven to be inapplicable for quantitative analysis of PVP surface concentration. Even so, the films based on CH-PVP with a low χ_SUB_ show an outsize adsorption of FBS protein which is probably promoted by preadsorbed PVP coils.

## 4 Conclusions

The layer-by-layer films have been obtained from chitosan graft copolymers with either PVP or PVA side chains alternated with dextran sulfate. The copolymers with the molecular weight of glucosamine backbone of 450 kDa, side chains of PVP of 2.4 kDa or PVA of 2.0 kDa and the degree of amino groups substitution χ_SUB_ as high as ∼0.33 were used to assemble the 1 or 5.5 bilayer films with a bilayer thickness in the range from 1.3 to 4.2 nm. The content of water in the film material reaches 85 ± 5% with virtually no effect of the structure of the copolymer.

An analysis of the protein repellency of LbL coatings based on graft copolymers of chitosan with hydrophilic oligomers, reveals that the behavior of multilayer coatings based on graft copolymers is common for PEG, dextran, PVA, and PVP side chains and is determined by the average distance between hydrophilic macromolecules on the LbL film surface. Regardless of the structure of the side chain of the copolymer, a decrease in the adsorption of proteins on the surface of multilayer coatings occurs as side chains begin to overlap. The minimum protein adsorption is observed if average interchain distance is close to radius of gyration of side chain coils.

## Declarations

### Author contribution statement

Kanstantsin S. Livanovich: Conceived and designed the experiments; Performed the experiments; Analyzed and interpreted the data; Wrote the paper.

Anastasiya A. Sharamet: Performed the experiments; Analyzed and interpreted the data; Contributed reagents, materials, analysis tools or data.

Anna N. Shimko: Conceived and designed the experiments; Analyzed and interpreted the data; Contributed reagents, materials, analysis tools or data.

Tatsiana G. Shutava: Conceived and designed the experiments; Performed the experiments; Analyzed and interpreted the data; Wrote the paper.

### Funding statement

This work was supported by grant 2.2.2.5 from the State Scientific Research Programs for 2021-2025, the Republic of Belarus.

### Data availability statement

Data included in article/supplementary material/referenced in article.

### Declaration of interests statement

The authors declare no conflict of interest.

### Additional information

No additional information is available for this paper.
