# Supplementary material for: Layer-by-layer films of polysaccharides modified with poly(N-vinylpyrrolidone) and poly(vinyl alcohol)
Source: Heliyon. 2021 Oct 21;7(10):e08224. doi: 10.1016/j.heliyon.2021.e08224 (PMC8555284; doi:10.1016/j.heliyon.2021.e08224)
Supplement: Supplementary file 1 — Supplementary material [file mmc1.docx]

Supplementary Material

Layer-by-Layer Films of Polysaccharides Modified with Poly(N-vinylpyrrolidone) and Poly(vinyl alcohol)

Kanstantsin S. Livanovich^1^*, Anastasiya A. Sharamet^1^, Anna N. Shimko^2^, Tatsiana G. Shutava^1^

^1^ Institute of Chemistry of New Materials, National Academy of Sciences of Belarus, Minsk, Belarus;

^2^ Center for analytical spectral measurements, B.I. Stepanov Institute of Physics, National Academy of Sciences of Belarus, Minsk, Belarus

*S1. Preparation of PVP 2.4 kDa*

Ethanol (3 mL) is placed in a three-necked flask equipped with a magnetic stirrer, reflux condenser, dropping funnel and argon supply tube. Argon is bubbled through the solvent under vigorous stirring for 10 min. 0.1 mol (10.7 mL) of N-vinylpyrrolidone (VP) is added to the flask. After 10 min 1.3·10^-4^ mol of 3-mercaptopropionic acid (MPA) as a 0.13 M solution in ethanol and 1 mL of an alcohol solution of 4,4'-azobis (4-cyanovaleric acid) (ABCV) with the concentration of 0.089 M (25 mg, 8.9 10^-5^ mol). The reaction mixture is placed in a water bath at 70 ± 1 °C for 5 min. Then a 0.31 M alcohol solution of MPA is fed into the stirring mixture at a rate of 2.5 mL/h for 6 h. The polymerization process is stopped by cooling the reaction mixture to room temperature. After completing the synthesis, the solvent and monomer are distilled off on a rotary evaporator at 70 ° C. The polymer is dissolved in 20 mL of acetone and precipitated by the addition of 40 mL of diethyl ether, the mother liquor is discarded. The precipitate is redissolved in 10 mL of acetone and precipitated with 20 mL of ether. The cleaning procedure is repeated 4 times. The resulting polymer is dried under vacuum to constant mass.

*S2 Preparation of PVA 2.0 kDa*

In a three-necked flask equipped with a magnetic stirring bar, reflux condenser, dropping funnel and argon supply tube, 9 mL of ethanol is placed and bubbled with argon for 10 min under vigorous stirring. 0.25 mol of vinyl acetate is added to the system and argon is bubbled through the system for another 10 min. 1 mL of 0.1 M alcoholic solution of MPA is added into the flask followed by 1 mL of a 0.089ºM solution of ABCV (25 mg, 8.9 10^-5^ mol). The reaction mixture was placed in a water bath at 70 °C for 5 min. An 0.23 M MPA solution in ethanol is fed into the reaction mixture with stirring at a rate of 2.5 mL/h for 6 h. The polymerization process is stopped by cooling the reaction mixture to room temperature. After completing the synthesis, the solvent and monomer are distilled off on a rotary evaporator at 70 ° C. The polymer is dissolved in 20 mL of acetone, transferred to a 150 mL separatory funnel and 50 mL of hexane is added. After separation of the phases, the lower polymer phase is separated, the upper phase is discarded. The polymer solution is diluted with 10 mL of acetone, transferred to a separatory funnel and 25 mL of hexane is added. Thus, the phase separation procedure is repeated 4 times. The solvent is distilled off from the polymer solution on a rotary evaporator at 70 °C and dried under vacuum to constant weight.

In order to obtain poly(vinyl alcohol) of 2.0 kDa polyvinyl acetate is hydrolyzed in acidic medium. A three-necked flask equipped with a magnetic stirrer and a reflux condenser is charged with a 0.3 g/mL solution of poly(vinyl acetate) in ethanol. With stirring, a solution of sulfuric acid weighing 1.84 g in 3 mL of ethanol is introduced into the flask and the mixture is slowly heated for 30 min to 70 °C. The reaction mixture is kept at this temperature for 6 h. By the end of the reaction, the content of the flask is cooled to room temperature, precipitated poly(vinyl alcohol) is filtered through a glass filter funnel under reduced pressure, the precipitate is washed 4-6 times on the filter with ethanol until pH of washing alcohol is neutral, then 2 times with diethyl ether. The obtained PVA is dried under vacuum to constant weight.

*S3. FTIR spectroscopy of synthesized PVA*





Figure S1. FTIR spectrum of synthesized PVA.

*S4. Synthesis of CH-PVP and CH-PVA with different χ_SUB_*

A 10 mg/mL CH 450 kDa solution and a 50 mg/mL SUB (PVP 2.4 kDa or PVA 2.0 kDa) solution in 0.1 M MES buffer (pH 6.1) supplemented with 0.2 M NaCl were prepared. An aliquot (150 μL) of 5M hydrochloric acid was added to increase solubility of CH. The CH and SUB solutions were mixed in accordance with Table A and Table B. pH after mixing is ranged from 5.4 to 5.6. EDC was dissolved in 0.75 mL of the buffer and added to each polymer mixture. The volume of the mixture (V_Σ_) was adjusted with distilled water. The reaction mixture was stirred for 15 h at 20 ±1 ^0^C.

Table S1. Mixing ratio of CH, SUB and EDC for CH-PVP preparation

| χ, mol/mol | | V, mL | | m, mg | | | V_Σ_, mL |
| --- | --- | --- | --- | --- | --- | --- | --- |
| Theory | Experiment | CH | PVP | CH | PVP | EDC |  |
| 0.01 | 0.01* | 6 | 0.2 | 60 | 10 | 7.5 | 11 |
| 0.05 | 0.05* | 5 | 0.8 | 50 | 40 | 30 | 11 |
| 0.1 | 0.12 | 5 | 1.5 | 50 | 75 | 30 | 15 |
| 0.2 | 0.206 | 5 | 3.0 | 50 | 150 | 60 | 15 |
| 0.3 | 0.305 | 5 | 4.5 | 50 | 225 | 90 | 15 |

* calculated from the mass of copolymer obtained after the synthesis

Table S2. Mixing ratio of CH, SUB and EDC for CH-PVA preparation

| χ, mol/mol | | V, mL | | m, mg | | | V_Σ_, mL |
| --- | --- | --- | --- | --- | --- | --- | --- |
| Theory | Experiment | CH | PVA | CH | PVA | EDC |  |
| 0.01 | 0.01* | 8.5 | 0.24 | 85 | 12 | 20 | 12 |
| 0.05 | 0.05* | 7.5 | 1 | 75 | 50 | 50 | 12 |
| 0.1 | 0.132 | 6.1 | 2.0 | 61 | 80 | 40 | 15 |
| 0.2 | 0.173 | 5 | 3.25 | 50 | 130 | 60 | 15 |
| 0.3 | 0.33 | 4.4 | 4.3 | 44 | 170 | 80 | 15 |

*S5. QCM experiment*


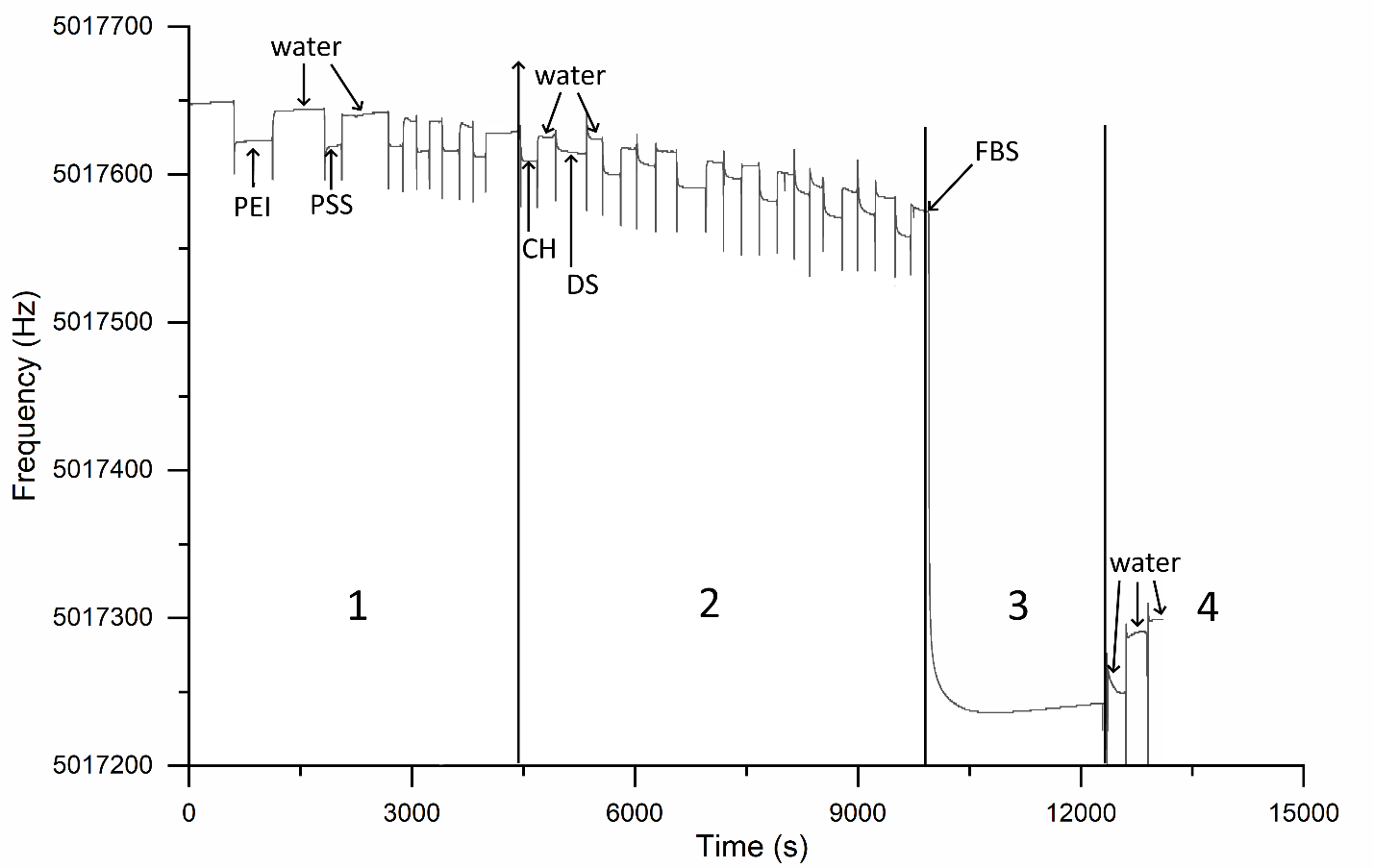


Figure S2. Typical frequency change of the QCM resonator during experiment: (PEI/PSS)_3_ precursor film (1); formation of (CH/DS)_5.5_ film (2); FBS solution exposure (3); washing stage (4).

*S6. Dry mass of (CH-PVA/DS)_n_ films*





Fig S3. Dependence of calculated dry mass (m_d_) of (CH-PVA/DS)_n_ films with different copolymer substitution degree (χ_PVA_) on the number of bilayers.

*S7. Absorbance of FITC in aqueous solution of PVP*





a b

Fig S4. Spectra of a 1 µg/mL FITC solutions in the phosphate buffered saline (pH 7.4) solutions containing different mass percent concentration of PVP (a), and the dependency of FITC absorbance at 496 nm from PVP mass percent concentration (b).
